# Insecticidal Activity of Plant Essential Oils Against the Vine Mealybug, *Planococcus ficus*

**DOI:** 10.1673/031.013.14201

**Published:** 2013-12-04

**Authors:** Filitsa Karamaouna, Athanasios Kimbaris, Αntonios Michaelakis, Dimitrios Papachristos, Moschos Polissiou, Panagiota Papatsakona, Eleanna Tsora

**Affiliations:** 1 Benaki Phytopathological Institute, Department of Pesticides' Control and Phytopharmacy, Department of Entomology & Agricultural Zoology, 8 St. Delta str., 145 61 Kifissia; 2 Democritus University of Thrace, Faculty of Agricultural Development, 193 Pantazidou Street, 68200 New Orestiada, Greece; 3 Benaki Phytopathological Institute, Department of Entomology & Agricultural Zoology, 8 St. Delta str., 145 61 Kifissia; 4 Laboratory of Chemistry, Department of Science, Agricultural University of Athens, Iera Odos 75, 11855, Athens, Greece

**Keywords:** basil, botanical insecticides, citrus, lavender, mealybugs, mint, thyme—leaved savory, toxic effect

## Abstract

The vine mealybug, *Planococcus ficus* (Signoret) (Hemiptera: Pseudococcidae), is a pest in grape vine growing areas worldwide. The essential oils from the following aromatic plants were tested for their insecticidal activity against *P. ficus*: peppermint, *Mentha piperita* L. (Lamiales: Lamiaceae), thyme-leaved savory, *Satureja thymbra* L., lavender, *Lavandula angustifolia* Mill, and basil, *Ocimum basilicum* L. Essential oils from peels of the following fruits were also tested: lemon, *Citrus limon* L. (Sapindales: Rutaceae), and orange, *C. sinensis* L. The reference product was paraffin oil. Bioassays were conducted in the laboratory by using spray applications on grape leaves bearing clusters of *P. ficus* of one size class, which mainly represented either 3rd instar nymphs or pre-ovipositing adult females. The LC_50_ values for each essential oil varied depending on the *P. ficus* life stage but did not significantly differ between 3^rd^ instar nymphs and adult females. The LC_50_ values of the citrus, peppermint, and thyme-leaved savory essential oils ranged from 2.7 to 8.1 mg/mL, and the LC_50_ values of lavender and basil oil ranged from 19.8 to 22.5 and 44.1 to 46.8 mg/mL, respectively. The essential oils from citrus, peppermint and thymeleaved savory were more or equally toxic compared to the reference product, whereas the lavender and basil essential oils were less toxic than the paraffin oil. No phytotoxic symptoms were observed on grape leaves treated with the citrus essential oils, and low phytotoxicity was caused by the essential oils of lavender, thyme-leaved savory, and mint, whereas the highest phytotoxicity was observed when basil oil was used.

## Introduction

Plant essential oils (EOs) exhibit biological activity against a wide spectrum of plant pests and may act as fumigants, contact insecticides, repellents, and antifeedants, or they can affect the growth rate, reproduction, and behavior of insect pests (Harwood et al. 1990; Isman 2000; Papachristos and Stamopoulos 2002a, 2004; Petrakis et al. 2005; Isman et al. 2008). In addition, the low mammalian toxicity of EOs and their rapid degradation in the environment make them attractive alternatives compared to conventional pesticides (Rebenhorst 1996; Misra and Pavlostathis 1997; Isman 2000).

The EOs of the aromatic plants of mint species (*Mentha* spp.) have adulticidal, larvicidal, and growth and reproduction inhibitory effects, as well as repellent activity against various stored product pests and vectors (Rajendran and Sriranjini 2008; Kumar et al. 2010, 2011; Michaelakis et al. 2011). They also exhibit strong fumigant toxicity against green house pests such as *Trialeurodes vaporariorum*, *Tetranychus urticae* (Choi et al. 2003, 2004), and several aphids species (Kimbaris et al. 2010). Lavender (*Lavandula* spp.) EOs have fumigant toxicity, reproduction inhibitory effects, and repellent action against the bean weevil, *Acanthoscelides obtectus* (Papachristos and Stamopoulos 2002a, b, 2004), and repellent, toxic, and oviposition deterrent effects against *T. urticae* and *Eutetranychus orientalis* (Refaat et al. 2002). Citrus EOs have been found to participate in resistance of citrus fruits against infestation of Mediterranean fruit fly, *Ceratitis capitata* (Papachristos et al. 2009), and to posses fumigant and contact insecticidal properties against a high range of stored product and agricultural pests as well as disease vector pests (Don-Pedro 1996; Hollingsworth 2005; Michaelakis et al. 2009; Kimbaris et al. 2010). Basil, *Ocimum basilicum* L. (Lamiales: Lamiaceae), EOs have been exclusively tested against a range of insects and mite pests of crops and stored products, exhibiting contact and fumigant toxicity that affects the development and behavior of insect pests (Papachristos and Stamopoulos 2002a; Refaat et al. 2002; Pascual-Villalobos and Ballesta-Acosta 2003; Yi et al. 2006; Kostić et al. 2008; Chang et al. 2009).

The vine mealybug, *Planococcus ficus* (Signoret) (Hemiptera: Pseudococcidae), is a key pest in grapevine growing areas worldwide, such as Mediterranean regions of Europe, North and South Africa, the Middle East, California, Mexico, and Argentina) (Dalla Monta et al. 2001; Varikou et al. 2010). It infests table and wine grapes and raisins, lowering the crop quality by feeding on the grape bunches and by excreting honeydew, which acts as a substrate for sooty mold (Walton and Pringle 2004, 2005). It also reduces the vine vigor by feeding on all parts of the plant (roots, trunk, cordons, canes, leaves) (Haviland et al. 2005). Furthermore, *P. ficus* is a vector of the grapevine leafroll associated virus 3 (Engelbrecht and Kasdorf 1990) and is therefore considered economically important even at low densities (Gülec et al. 2007).

On nursery stock, the control of *P. ficus* is considered critical to prevent further dissemination of the pest in non-infested grape growing regions. The treatment of dormant nursery grape cuttings is currently based on hot-water immersions, which can be laborintensive and expensive. Also, excessive high temperatures can cause plant damage. Controlled atmosphere treatments with ultralow oxygen have also been investigated, with encouraging results on the efficacy and with no adverse effects on plant growth (Haviland et al. 2005; Yong-Biao Liu et al. 2010).

Although a combination of chemical, biological and cultural control methods may be used in an integrated pest management system to control vine mealybug in vineyards today, chemical insecticide applications using synthetic products are the most common control method against the pest (Daane et al. 2003; Walton 2003; Daane et al. 2004; Castillo et al. 2005; Gülec et al. 2007). However, chemical control programs of *P. ficus* are often incomplete, as a portion of the population often resides in protected locations under the bark of the trunk or cordon, and can disrupt natural enemies (Daane et al. 2003, 2004; Walton 2003; Castillo et al. 2005; Desneux et al. 2007; Gülec et al. 2007). Moreover, chemical insecticide programs against the pest in the USA, including organophosphates combined with either or both a systemic chloronicotinyl and an insect growth regulator, can be prohibitively expensive for some grape producers (Daane at al. 2004). Only a few insecticides (mainly paraffin oils) are currently registered for control of *P. ficus* in the European Union, posing a risk for resistance development by their repeated use (Hellenic Ministry of Rural Development and Food 2012; MAPA 2012; MiPAAF 2012). A number of adverse ecological and health impacts from intensive use of synthetic pesticides in crop protection (Isman 2006) highlight limitations of the chemical control of the pest.

Having in mind the above restrictions, botanical insecticides, including plant EOs, are generally considered potential alternatives to synthetic insecticides (Isman 2006), but few data exist on their effect on mealybugs. Hollingsworth (2005) found a high insecticidal effect of limonene, the major constitute of citrus fruit EOs, on some mealybug species. Cloyd and Chiasson (2007) and Cloyd et al. (2009) evaluated the toxic effects of some commercially available plant products, including EOs, with promising results.

The aim of this study was to examine the insecticidal activity of EOs extracted from four aromatic plants and two citrus fruits against *P. ficus*. In addition, as some essential oils exhibit phytotoxic action (Tworkoski 2002), phytotoxic effects of the EOs on grape leaves were assessed. The results should give an insight into the potential use of the tested EOs as botanical insecticides and advance the development of alternative effective synthetic insecticides against this pest in nurseries and vineyards.

## Materials and Methods

### Plant material and isolation of the essential Oils

Plant EOs were selected based on their toxicity against other insect pests (Harwood et al. 1990; Don-Pedro 1996; Papachristos and Stamopoulos 2002a, b; Yi et al. 2006; Kimbaris et al. 2010; Michaelakis et al. 2011). Samples were collected from the aerial parts of the aromatic plants, thyme-leaved savory, *Satureja thymbra* L. (Lamiales: Lamiaceae), lavender, *Lavandula angustifolia* L., peppermint, *Mentha piperita* L., and *O. basilicum*, and the fruit peels of lemon, *Citrus limon* L. (Sapindales: Rutaceae), and orange, *C. sinensis* L. growing in native habitats throughout Greece. Basil, thyme-leaved savory, lavender, and peppermint (cultivated plants) were collected in Karditsa (central Greece) between July and August 2009. Orange and lemon fruits were collected in Arta (Epirus, central-western Greece) in mid March 2008.

For the aromatic plants, all four materials were air-dried, aerial parts were powdered in an electric blender, and 0.5 kg of each sample was hydrodistilled. For lemon and orange, 0.5 kg of fresh fruit peels were used for hydrodistillation. The EOs were obtained by the hydrodistillation of plant samples by using a Clevenger-type apparatus for 3 hr at 100° C. Samples were then dried over anhydrous magnesium sulphate, filtered, and stored in a freezer at -22° C until analyzed and used. Their volume was calculated and expressed as mL of EO/100 g of dry (aromatic plants) or fresh (orange, lemon, fruit peels) material.

### Chemical standards

*R*-(+)-pulegone, *α*-pinene, *β*-pinene, thymol, carvacrol, myrcene, *p*-cymene, citral (mixture of neral and geranial), neryl acetate, caryophyllene oxide, geranyl acetate, (-)- menthol, terpinen-4-ol, eugenol, geraniol, eucalyptol, *S*-(-)-limonene, terpinolene, borneol, camphor, *γ*-terpinene, linalool, *β*- caryophyllene, methyl chavicol, *α*-terpineol, linalool acetate, *δ*-2-carene, nerol, *β*- ocimene, and (+)-menthone were purchased from Sigma-Aldrich (www.sigmaaldrich.com). Piperitone and *iso*-menthone were purchased from Extra Synthese (www.extrasynthese.com). Purities of all the above standards were more than 97% except *β*-ocimene (90%), which was given as a mixture of the *Z* and *E* isomers with limonene. Diethyl ether was purchased from SDS (www.carloerbareagents.com).

### Gas chromatography-mass spectrometry

The EOs were analyzed using a Hewlett Packard II 5890 gas chromatography system (www.hp.com), equipped with a FID detector and HP-5ms capillary column (30 m × 0.25 mm, film thickness 0.25 µm). Injector and detector temperatures were set at 220o C and 290o C, respectively. Gas chromatography oven temperature was programmed to increase from 60° C to 240° C at a rate of 3° C/min and then held isothermally for 10 min. Helium was used as the carrier gas at a flow rate of 1 mL/min. Diluted samples of 1 µL (1/100 in diethyl ether, v/v) were injected manually in the splitless mode. Quantitative data were obtained electronically from the FID area without the use of correction factors. Gas chromatography/mass spectrometry analysis was performed under the same conditions as gas chromatography using the same Hewlett Packard instrument equipped with a Hewlett Packard II 5972 mass selective detector in the electron impact mode (70 eV). Injector and mass spectrometry transfer line temperatures were set at 220° C and 290° C, respectively. The most abundant constituents as well as some minor constituents of the EOs were identified by comparing gas chromatography relative retention times and mass spectra with those of pure standards. Tentative identification of the remaining components was based on the comparison of their mass spectra and elution order with those obtained from the NIST 98 and Wiley 275 libraries (Adams 2007).

### Host plant-mealybug primary cultures

The host plant was *Vitis vinifera* L. (Vitales: Vitaceae) cv. Soultanina grown in 20-L pots. The primary culture of *P. ficus* was established in the insectary of the Benaki Phytopathological Institute (Laboratory of Biological Control of Pesticides) from individuals collected from an infested vineyard in the region of Helia-Peloponnese. Taxonomic identification of the species was done according to Cox and Ben-Dov (1986) key. The culture was maintained on sprouted potatoes in sandwich boxes (17 × 11 × 5 cm, Length × Width × Height) with two net covered openings (diameter = 1.5 cm) at the sides for ventilation. The boxes were kept in a Gallenkamp CΟ2 growth chamber at 26° C and constant dark. All biological stages of *P. ficus* were present in the culture.

### Bioassays

Bioassays were conducted under laboratory conditions in Petri dishes (diameter = 9 cm) that had lids with openings (diameter = 6 cm) covered with fine muslin for ventilation. Grape leaves of approximately the same size were placed on a layer of agar in the Petri dishes. Two size classes of *P. ficus*, 1–1.5 mm and > 1.5 mm (mainly 3^rd^ instar nymphs and pre-ovipositing adult females respectively), were tested. Mealybugs of the same size class (life stage) were placed on the grape leaves in the Petri dishes a few hours prior to the bioassays.

The Petri dishes containing the leaf with *P. ficus* were sprayed with 1 mL aqueous solution of the EOs (using 1% Tergitol as an emulsifier). Spraying was carried out using a small volume vessel of pharmaceutical use with a spray vaporisateur. The excess run off solution was removed from the Petri dishes immediately after spraying, and the dishes were then covered with the lids bearing the ventilation holes to prevent vapor accumulation. The same procedure was followed for the control group, which consisted of a) water, b) water with 1% Tergitol, and c) a reference product. The reference product was the paraffin oil Triona 81 EW (paraffin oil 81% w/w), which is registered in Greece against the red mite, *Panonychus ulmi*, in grapes and the citrus mealybug, *Planococcus citri*, in citrus, pome fruits, and stone fruits. Three to four concentrations (mg of essential or paraffin oil/mL aqueous Tergitol) were tested for each essential/paraffin oil and *P. ficus* life stage: 4.5, 9, and 13.5 mg/mL for orange; 0.9, 4.5, 9, and 13.5 mg/mL for lemon; 4.5, 9, 13.5, and 18 mg/mL for thymeleaved savory; 4.5, 9, and 18 mg/mL for peppermint; 9, 18, 27, and 36 mg/mL for lavender; 9, 36, 45, and 63 mg/mL for basil; and 6.4, 12.8, 19.2, and 25.9 mg/mL for paraffin oil. Each concentration was tested three to four times (replications). Twenty-four hours after application, insect mortality was recorded and the sprayed leaves were checked for the presence of phytotoxicity. When phytotoxic effects were observed (namely discoloration and necrosis of the leaf), evaluation of phytotoxicity severity was assessed as leaf surface percentage with symptoms, i.e., brown necrotic spots or discoloration over a wider area of the leaf blade: none (0–1%), slight (1–25%), medium (25–50%), high (> 50%).

### Data analysis

Mortality data obtained from each doseresponse trial were subjected to probit analysis and LC_50_ and LC_90_ values and 95% confidence intervals were estimated. LC50 or LC_90_ values were compared using respective confidence intervals (Finney 1971). Statistical analysis was conducted using SPSS 14.0 (SPSS 2005).

**Table 1.**
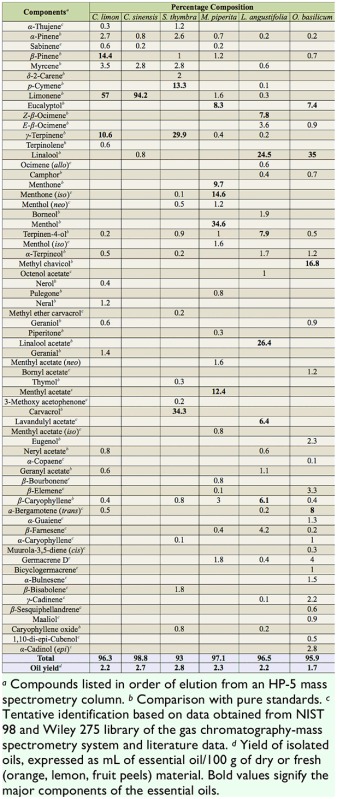
Chemical composition (%) of essential oils derived from two citrus species (*Citrus limon*, and *C. sinensis*) and four aromatic plant species (*Satureja thymbra*, *Mentha piperita*, *Lavandula angustifolia*, and *Ocimum basilicum*).

## Results

### Chemical composition of plant oils

The qualitative and quantitative compositions of the EOs of the aromatic plants and fresh citrus fruit peels are shown in Table 1. Focusing on the most abundant ingredients of the aromatic plant oils, basil consisted primarily of linalool and methyl chavicol. The thyme-leaved savory oil contained carvacrol and terpinene. The main components of lavender oil were linalool acetate and linalool. The peppermint oil consisted of menthol in high percentage, menthone (*iso*), *β*-pinene, and menthyl acetate. In the citrus oils, limonene was by far the most abundant ingredient and it was present in a higher percentage in orange than in lemon.

### Effect of plant essential oils on *P. ficus*

The LC_50_ values of citrus, mint and thymeleaved savory oils ranged from 2.7 to 8.1 mg/mL depending on the EO and the mealybug life stage (Table 2). These LC50 values were significantly lower than the LC_50_ of the reference paraffin oil in the respective *P. ficus* life stages, and therefore indicate a higher toxic effect of the tested ΕΟs compared to the reference product. The LC_50_ values for each EO did not reveal any significant differences between nymphs and adults (Table 2).

### Phytotoxicity of essential oils on grape Vine

**Figure 1.**
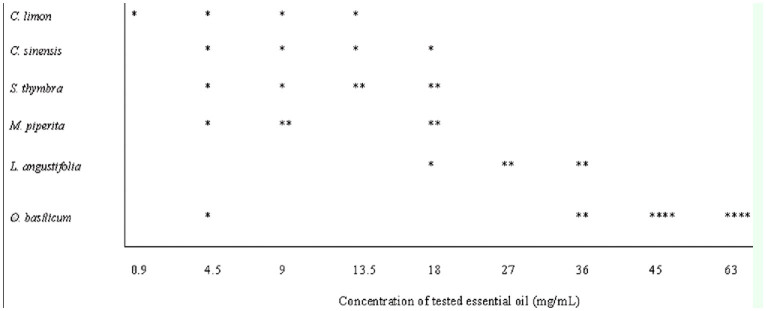
Phytotoxicity of essential oils derived from two citrus species (*Citrus limon* and *C. sinensis*) and four aromatic plant species (*Satureja thymbra*, *Mentha piperita*, *Lavandula angustifolia*, and *Ocimum basilicum*) on grape leaves. Evaluation of phytotoxicity severity was assessed as leaf surface percentage with symptoms: none (0–1%), slight (1–25%), medium (25–50%), and high (> 50%), indicated with *, **, *** and ****, respectively. High quality figures are available online.

No phytotoxic symptoms were observed on grape leaves treated with the citrus oils, the aqueous Tergitol solution, or the reference paraffin oil. The leaves sprayed with the EOs from the aromatic plants developed brown spots, which later became necrotic. The EOs of lavender (> 27 mg/mL), thymeleaved savory (> 13.5 mg/mL), mint (> 9 mg/mL), and paraffin oil (25.9 mg/mL) caused slight phytotoxicity (leaf surface percentage with symptoms did not exceeded 25%), whereas basil EO caused high phytotoxicity (leaf surface percentage with symptoms over 50%) in most of the concentrations applied (Figure 1).

**Table 2.**
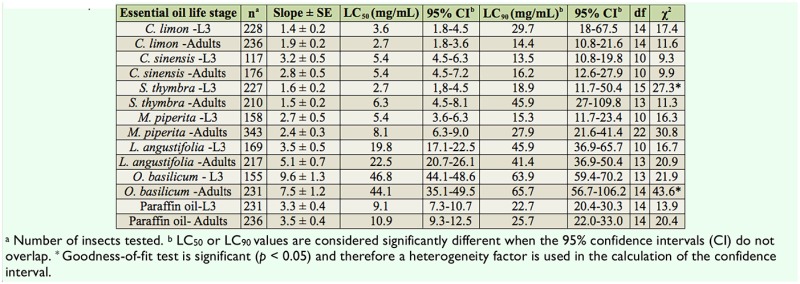
LC_50_ and LC_90_ (mg/mL) of plant essential oils derived from two citrus species (*Citrus limon* and *C. sinensis*) and four aromatic plant species (*Satureja thymbra*, *Mentha piperita*, *Lavandula angustifolia*, and *Ocimum basilicum*) against 3^rd^ instar nymphs and female adults of *Planococcus ficus*.

## Discussion

The results on the composition of the aromatic plant and citrus EOs are in agreement with literature data on other pests (Goren et al. 2004; Hassiotis et al. 2010; Kimbaris et al. 2010; Ntalli et al. 2010). The insecticidal activity of the tested EOs on *P. ficus* differed, but overall EOs were equally toxic to 3^rd^ instar nymphs and adults. The citrus peel EOs (mainly limonene) were the most toxic of all the tested EOs, whereas the lavender and basil oil possessed the lowest insecticidal activity among the EOs and the reference paraffin oil. Other comparative toxicity studies of EO vapours of orange mainly limonene), peppermint (consisting mainly of *p*-menthane), and basil (consisting mainly of linalool, methyl chavicol, eucalyptol) on several aphid species reported these EOs as highly toxic, with peppermint oils being the most toxic and orange oils being the least toxic (Kimbaris et al. 2010).

The insecticidal activity of pure limonene has been shown against other mealybug species. Spray applications of limonene in 1% aqueous solution (together with a spray adjuvant as an emulsifier/surfactant and another agricultural surfactant) resulted in 44% mortality of 3rd and 4^th^ instar nymphs of *P. citri* on gardenia pot plants in the greenhouse. This mortality was equal to the mortality caused by a reference insecticidal soap (49% potassium salts of fatty acids) and significantly higher than the mortality caused by a reference horticultural spray oil (98.8% paraffinic oil) (Hollingsworth 2005). In addition, laboratory bioassays with the same limonene solution showed 95–100% mortality to the nymphs and adults of the coconut mealybug *Nipaecoccus nipae*, on sprayed coconut leaves, 92% mortality against 3^rd^ and 4^th^ instar nymphs of the longtailed mealybug, *Pseudococcus longispinus*, when applied on green beans by dipping for 1 min, and 100% mortality of eggs of the root mealybug, *Rhizoecus* spp., on Gardenia roots by dipping in application for 1 min (Hollingsworth 2005).

The citrus EOs did not cause phytotoxicity on grape leaves. EOs of the aromatic plants applied in concentrations that were toxic against *P. ficus* were also phytotoxic. Selectivity data of 1% limonene in aqueous solution showed phytotoxic effects on ferns, gingers, and delicate flowers but caused no damage to ornamentals with thick, waxy leaves, such as palms, cycads, and orchids (Hollingsworth 2005). In addition, limonene has been shown to be phytotoxic to strawberries at concentrations exceeding 3% and cabbage and carrot seedlings at concentrations higher than 9% (Ibrahim et al. 2001). Hollingworth (2005) has pointed out the significance of a good emulsion when mixing an oil (such as limonene) with a solution of an emulsifier in order to prevent the two phases of the formulation from breaking and separating more readily, which would consequently result in variability of the EO insecticidal effect on the target pest and cause phytotoxicity on the plant. Improved formulations of the tested EOs (especially from the aromatic plants) may enhance their effectiveness and reduce their phytotoxic effect.

Overall, the high insecticidal activity and lack of any phytotoxic effect on grape vine by citrus oils suggest that lemon and orange peels are the most attractive botanical sources among the tested EOs for the production of alternative plant protection products against *P. ficus*. This is even more so given that the extraction process of the citrus oils is simple and cost effective, as they could be produced as byproducts from the juice industry. The present study provides a first screening on the insecticidal activity of the tested EOs on *P. ficus*. Excessive experimentation is necessary to determine the efficacy of the EOs in semifield and eventually field conditions and possible adverse effects on grapes and the natural enemies of *P. ficus*. Whether a stronger insecticidal effect of the citrus EOs versus the aromatic plant EOs on *P. ficus* means a stronger adverse effect on its Coccinelid predators will have to be investigated, as will the eco-toxicological profile of the EOs.

## Abbreviations

**EO**,: essential oil
